# Novel antibodies to phosphorylated α-synuclein serine 129 and NFL serine 473 demonstrate the close molecular homology of these epitopes

**DOI:** 10.1186/s40478-016-0357-9

**Published:** 2016-08-08

**Authors:** Nicola J. Rutherford, Mieu Brooks, Benoit I. Giasson

**Affiliations:** 1 Department of Neuroscience, College of Medicine University of Florida, Gainesville, FL 32610 USA; 2 Center for Translational Research in Neurodegenerative Disease, College of Medicine University of Florida, Gainesville, FL 32610 USA; 3 McKnight Brain Institute, College of Medicine University of Florida, Gainesville, FL 32610 USA

**Keywords:** Monoclonal antibodies, α-synuclein, Neurofilament, Parkinson’s disease, Phosphorylation

## Abstract

Pathological inclusions containing aggregated, highly phosphorylated (at serine129) α-synuclein (αS pSer129) are characteristic of a group of neurodegenerative diseases termed synucleinopathies. Antibodies to the pSer129 epitope can be highly sensitive in detecting αS inclusions in human tissue and experimental models of synucleinopathies. However, the generation of extensively specific pSer129 antibodies has been problematic, in some cases leading to the misinterpretation of αS inclusion pathology. One common issue is cross-reactivity to the low molecular mass neurofilament subunit (NFL) phosphorylated at Ser473. Here, we generated a series of monoclonal antibodies to the pSer129 αS and pSer473 NFL epitopes. We determined the relative abilities of the known αS kinases, polo-like kinases (PLK) 1, 2 and 3 and casein kinase (CK) II in phosphorylating NFL and αS, while using this information to characterize the specificity of the new antibodies. NFL can be phosphorylated by PLK1, 2 and 3 at Ser473; however CKII shows the highest phosphorylation efficiency and specificity for this site. Conversely, PLK3 is the most efficient kinase at phosphorylating αS at Ser129, but there is overlay in the ability of these kinases to phosphorylate both epitopes. Antibody 4F8, generated to the pSer473 NFL epitope, was relatively specific for phosphorylated NFL, however it could uniquely cross-react with pSer129 αS when highly phosphorylated, further showing the structural similarity between these phospho-epitopes. All of the new pSer129 antibodies detected pathological αS inclusions in human brains and mouse and cultured cell experimental models of induced synucleinopathies. Several of these pSer129 αS antibodies reacted with the pSer473 NFL epitope, but 2 clones (LS3-2C2 and LS4-2G12) did not. However, LS3-2C2 demonstrated cross-reactivity with other proteins. Our findings further demonstrate the difficulties in generating specific pSer129 αS antibodies, but highlights that the use of multiple antibodies, such as those generated here, can provide a sensitive and accurate assessment of αS pathology.

**Electronic supplementary material:**

The online version of this article (doi:10.1186/s40478-016-0357-9) contains supplementary material, which is available to authorized users.

## Introduction

Synucleinopathies are a group of neurodegenerative diseases that are characterized by the presence of proteinaceous inclusions containing aggregated α-synuclein (αS) [1–4]. These inclusions can occur within neurons in the form of Lewy bodies (LBs), Lewy neurites and neuronal cytoplasmic inclusions (NCIs) [1–4], and also within oligodendrocytes (glial cytoplasmic inclusions; GCIs). Synucleinopathies include Parkinson’s disease (PD), dementia with Lewy bodies (DLB) and multiple system atrophy (MSA) [5, 6], and αS positive inclusions also occur as a secondary proteinopathy in 50–60 % of Alzheimer’s disease patients and in many other neurodegenerative disorders [7–15]. Within pathological inclusions αS is aberrantly, highly (~90 %) phosphorylated at serine 129 (pSer129), while in its normal native state it is only phosphorylated at ~4 %, making this post translational modification a useful marker of αS inclusions [16, 17]. Therefore, antibodies recognizing pSer129 αS can be used as sensitive tools to detect abnormally aggregated αS in human brain tissue, as well as in experimental animal and cell culture studies where αS inclusion formation can be induced to assess the pathological outcome of these abnormal aggregated forms of αS.

Given the extensive use of pSer129 αS antibodies to assess for the presence of pathological αS inclusions, it is important to generate and validate the specificity of these reagents. Indeed many pSer129 αS antibodies can cross-react with additional phosphorylated proteins and it has been difficult to generate antibodies that are highly specific [4, 18]. For example, pSer129 αS antibody, 81A [19] that had been used in many studies to document the formation of pathological αS inclusions in model systems of induced inclusion formation [20–23], was later shown to lead to the over-representation or misinterpretation of the inclusion formation due to its strong cross-reaction to the low molecular mass neurofilament subunit (NFL) phosphorylated at serine 473 [18]. This issue underscores the importance of using highly specific and well characterized antibodies.

Herein, we have generated a series of novel monoclonal pSer129 antibodies and characterized the relative preferential specificity of casein kinase (CK) II and polo-like kinases (PLK) 1, 2 and 3 to phosphorylate Ser129 in αS and Ser473 in NFL. Most of these new antibodies demonstrated some variable cross-reactivity with the pSer473 NFL epitope. However, we also determined that the previously generated antibody EP1536Y and the new antibodies LS4-2G12 and LC3-2C2 did not react with phosphorylated NFL, although EP1536Y and LC3-2C2 had a tendency to non-specifically react with cellular nuclei. We demonstrated that the new pSer129 αS antibodies could be used to detect pathological inclusions in patients with synucleinopathies, as well as in induced mouse and cell culture models of αS inclusion formation. Collectively, these resources will be highly valuable tools for the field to accurately assess and monitor αS inclusion formation.

## Materials and methods

### Mouse lines

All procedures were performed according to the NIH Guide for the Care and Use of Experimental Animals and were approved by the University of Florida Institutional Animal Care and Use Committee. BALB/c mice and αS null (αS KO) mice [24] were obtained from Jackson Laboratory (Bar Habor, ME). M20 and M83 transgenic mice overexpressing wild-type and A53T human αS respectively, were previously described [25] and non-transgenic/wild-type (WT) littermates were also used. Stereotaxic [18, 26, 27] and muscle injection procedures [28] were previously described. NFL null (NFL KO) mice were previously described [29] and kindly provided by Dr. Janice Robertson.

### Antibodies

Mouse anti-NFL antibody NR4 was obtained from Sigma-Aldrich (St. Louis, MO). Anti-pSer129 αS rabbit monoclonal antibody EP1536Y was obtained from Abcam (Cambridge, MA). Anti-human αS mouse monoclonal antibody Syn 204 was previously described [30]. pSer129/81A is a mouse monoclonal antibody that only reacts with αS when phosphorylated at Ser129 [19], but also cross-reacts with phosphorylated NFL [18]. Neuronal specific rabbit anti-βIII tubulin antibody (T2200) was obtained from Sigma-Aldrich (St. Louis, MO). Rabbit polyclonal antibody SNL-4 detects αS residues 2–12 and was previously described [30].

### Generation of new mouse monoclonal antibodies

Peptides (Table 1) designed over the region of interest were synthesized and purified by GenScript USA Inc (Piscataway, NJ). Lyophilized peptides were reconstituted in phosphate buffered saline (PBS) and conjugated to Imject Maleimide-Activated mcKLH (Thermo Scientific, Waltham, MA). Injection solutions were prepared by combining 100 μg KLH-conjugated peptide in 200 μl PBS with 100 μl of either Freunds complete adjuvant (1^st^ injection; Sigma Aldrich, St. Louis, MO) or Freunds incomplete adjuvant (subsequent injections; Sigma Aldrich, St. Louis, MO) and vortexing for 15 mins until emulsified. Female BALB/c mice (Jackson Laboratory, Bar Harbor, ME) were injected subcutaneously. A second subcutaneous injection was administered 3 weeks later. Six weeks following the initial injection, mice were boosted with an intraperitoneal (IP) injection of 100 μg KLH-conjugated peptide in PBS. Three days later, mice were euthanized and spleens were harvested using aseptic technique.

**Table 1:** Peptides designed to produce antibodies to pSer129 αS and pSer473 NFL

Peptide	Sequence	Target
pSer129short	***C***AYEMP(pS)EEGYQ	pSer129 αS
pSer129long	DNEAYEMP(pS)EEGYQDYE***C***	pSer129 αS
pSer473	***C***EAKDEPP(pS)EGEAEEE	pSer473 NFL

***C*** – cysteine residue added for conjugation to KLH, p – phospho-group

Mouse myeloma (Sp2/O-Ag14; ATCC, Manassas, VA) cells were maintained in high glucose (4.5 g/L) Dulbecco’s Modified Eagle Medium (DMEM) with 10 % NCTC 135 Media (Sigma Aldrich, St. Louis, MO), 20 % hybridoma grade fetal bovine serum (FBS; Hyclone, Logan, UT), 100 U/ml penicillin, 100 U/ml streptomycin, 2 mM L-glutamine, 0.45 mM pyruvate, 1 mM oxaloacetate, and 0.2 U/ml insulin at 37 °C and 8 % CO_2_. Spleens were gently homogenized in 5 % FBS/Hank’s balanced salt solution (HBSS; Lonza, Walkersville, MD) and centrifuged to pellet cells. The cell pellet was resuspended in red blood cell lysis buffer (Sigma Aldrich, St. Louis, MO) and diluted with HBSS after one minute. The cells were then washed twice by centrifuging at 100 × g for 10 mins and resuspended in HBSS. Sp2/O-Ag14 cells were also washed twice with HBSS. Five million Sp2/O-Ag14 cells were added to 50 million spleen cells and, after centrifuging at 100 × g for 10 mins onto a culture dish, fusion was induced with 50 % polyethylene glycol 1450 (PEG; Sigma Aldrich, St. Louis, MO). After washing with HBSS, cells were incubated in Sp2/O-Ag14 media at 37 °C with 8 % CO_2_ overnight. The next day, the cells were gently detached from the plate and distributed into 96 well plates with Sp2/O-Ag14 media/0.5 % hybridoma enhancing supplement (Sigma Aldrich, St. Louis, MO)/HAT selection supplement (Sigma Aldrich, St. Louis, MO).

### Hybridoma screening

All hybridoma clones were screened for reactivity to the injected peptide by enzyme-linked immunosorbent assay (ELISA). MaxiSorp plates (Thermo Scientific, Waltham, MA) were coated with 1 μg/ml peptide in PBS and blocked with 5 % FBS/PBS. Media from the hybridomas were applied to plates, which were then incubated at room temperature for 3 h. Next, the plates were washed with PBS, and incubated with goat anti-mouse secondary antibody conjugated to horse radish peroxidase (HRP; Jackson Immuno Research Labs, West Grove, PA) for 1 h at room temperature. Then, plates were washed and TMB substrates (Pierce, Rockford, IL) were applied until color changes were observed. Reactions were then quenched with 1 M HCl and absorbance was measured at 450 nm. Clones that were positive by ELISA were transferred to larger culture plates as needed. The positive clones were next screened by immunohistochemistry (IHC) of a human autopsy case with abundant αS pathology.

### Immunohistochemistry analyses

Paraffin embedded tissue from αS transgenic and WT mice are described in Table 2. Paraffin embedded, formalin fixed human brain tissue was obtained through the University of Florida Neuromedicine Human Brain Tissue Bank (summarized in Table 3). Sequential tissue sections were deparaffinized with xylenes, and sequentially rehydrated with graded ethanol solutions (100-70 %). Antigen retrieval was performed by incubating sections in 0.05 % Tween-20 in a steam bath for 30 mins. Endogenous peroxidase activity was quenched with 1.5 % hydrogen peroxide/0.005 % Triton X-100/PBS for 20 mins. Sections were blocked with 2 % FBS/0.1 M Tris, pH 7.6 then incubated with primary antibody overnight at 4 °C. Following washing with 0.1 M Tris, pH 7.6, sections were incubated with biotinylated horse anti-mouse IgG or biotinylated horse anti-rabbit IgG secondary antibody (Vector Laboratories, Burlingame, CA) diluted in 2 % FBS/0.1 M Tris pH 7.6 for 1 h. Next, sections were washed with 0.1 M Tris, pH 7.6, then incubated with streptavidin-conjugated HRP (VECTASTAIN ABC kit; Vector Laboratories, Burlingame, CA) diluted in 2 % FBS/0.1 M Tris pH 7.6 for 1 h. Sections were washed with 0.1 M Tris, pH 7.6, and then developed with 3, 3′diaminobenzidine (DAB kit; KPL, Gaithersburg, MD). Reactions were stopped by immersing the slides in 0.1 M Tris, pH7.6, and sections were counterstained with Mayer’s hematoxylin (Sigma Aldrich, St. Louis, MO). Next, sections were dehydrated with an ascending series of ethanol solutions (70–100 %) followed by xylenes, and coverslipped using cytoseal (Thermo Scientific, Waltham, MA).

**Table 2:** Summary of mouse tissue used for immunohistochemical analysis of novel antibodies

Mouse line	Treatment	Fixative	References
M83^+/−^	Intramuscular injection of αS fibrils (motor impaired/terminal)	150 mM NaCl/ 70 % ethanol	[28]
M83^+/−^	Intramuscular injection of αS fibrils (motor impaired/terminal)	Formalin	[28]
M83^+/−^	Cerebral injection of αS fibrils (3 months post-injection)	150 mM NaCl/ 70 % ethanol	[26]
M83^+/+^	Naïve (7 month old; no phenotype)	150 mM NaCl/ 70 % ethanol	
M83^+/+^	Naïve (10–12 month old; motor impaired/terminal)	150 mM NaCl/ 70 % ethanol	
M20^+/−^	Cerebral injection of αS fibrils (4 months post-injection)	150 mM NaCl/ 70 % ethanol	[27]
M20^+/−^	Cerebral injection of αS fibrils (4 months post-injection)	Formalin	[27]
WT	Naïve	150 mM NaCl/ 70 % ethanol

**Table 3:** Summary of human tissue used for immunohistochemical analysis of novel antibodies

Neuropathological diagnosis	Region(s) examined	Fixative	Number of cases
DLB	Cingulate cortex, Midbrain	Formalin	2
MSA	Pons, Cerebellum	Formalin	2
PD	Midbrain	Formalin	2

### Recombinant αS protein production and purification

Recombinant WT human or mouse αS or human αS with serine 129 mutated to alanine (S129A) were expressed in *Escherichia coli* (*E. coli*) BL21 (DE3)/RIL (Agilent Technologies, Santa Clara, CA) using the respective cDNA cloned into the bacterial expression plasmid pRK172, and purified as previously described [31, 32]. Protein concentrations were determined by bicinchoninic acid (BCA) assay using bovine serum albumin (BSA; Pierce, Rockford, IL) as a standard.

### Recombinant NFL protein production and purification

Recombinant WT mouse NFL or mouse NFL with serine 473 mutated to alanine (S473A) were expressed in *E. coli* BL21 (DE3)/RIL (Agilent Technologies, Santa Clara, CA), using the respective cDNA cloned in the bacterial expression plasmid pET-23d and purified as previously described [18]. Protein concentrations were determined by Bradford assay using BSA as a standard.

### Radioactive and non-radioactive kinase reactions

To determine which kinases could phosphorylate αS and NFL, and to what extent, we performed radioactive kinase reactions. NFL and S473A NFL were dialyzed in Tris or 4-(2-hydroxyethyl)-1-piperazineethanesulfonic acid (HEPES) overnight to remove the urea. Recombinant proteins (16.3 pmol; αS, S129A αS, NFL or S473A NFL) were incubated in 25 μl reactions at 30 °C for 8 h with 60 ng PLK2 (Life Technologies, Carlsbad, CA) or 20 ng CKII (New England Biolabs, Ipswich, MA), PLK1 or PLK3 (Life Technologies, Carlsbad, CA), 200 μM adenosine triphosphate (ATP) with γ-^32^P ATP (PerkinElmer, Waltham, MA) in manufacturer recommended buffers (CKII buffer: 50 mM Tris-HCl, pH7.5, 10 mM MgCl_2_, 0.1 mM ethylenediamietetraacetic acid (EDTA), 2 mM dithiothreitol (DTT), 0.01 % Brij 35; PLK1 buffer: 50 mM HEPES, pH7.5, 10 mM MgCl_2_, 2.5 mM DTT, 0.01 % Triton X-100; PLK2 buffer: 25 mM HEPES, pH7.5, 10 mM MgCl_2_, 5 mM MnCl_2_, 0.5 mM ethylene glycol-bis(β-aminoethyl ether)-N,N,N’,N’-tetraacetic acid (EGTA), 0.5 mM Na_3_VO_4_, 5 mM β-glycerophosphate, 2.5 mM DTT, 0.01 % Triton X-100; PLK3 buffer: 25 mM Tris, pH7.5, 10 mM MgCl_2_, 0.5 mM EGTA, 0.5 mM Na_3_VO_4_, 5 mM β-glycerophosphate, 2.5 mM DTT, 0.01 % Triton X-100). Reactions without protein for each kinase, and without kinase for each protein, were included as control reactions. Each reaction was performed in triplicate. Reactions were then absorbed onto P-81 phosphocellulose filters (GE Healthcare, Buckinghamshire, UK) and immersed in 85 mM phosphoric acid. After extensive washing in 85 mM phosphoric acid, the filters were rinsed with acetone, air dried and placed in scintillation vials with Econo-Safe Liquid Scintillation cocktail (Research Products International, Mount Prospect, IL). Radioactivity (^32^P) incorporation was measured using an LS5000 TD liquid scintillation counter (Beckman, Brea, CA).

CKII and PLK3 reactions for immunoblot antibody testing were performed as before, but without γ-^32^P ATP. Sodium dodecyl sulfate (SDS) sample buffer was added to the reactions which were incubated for 10 mins at room temperature. Equimolar amounts of protein were resolved by SDS-PAGE and analyzed by immunoblot.

### Sequential biochemical fractionation of mouse nervous tissue

Mouse nervous tissues were fractionated as described by Emmer et al. 2011 [33]. Briefly, mice were humanely euthanized, and the brain, brainstem and spinal cord were harvested. The cortex and brainstem/spinal cord were dissected and placed in separate tubes. Tissues were homogenized with 3 volumes per gram of tissue with high salt (HS) buffer (50 mM Tris, pH7.5, 750 mM NaCl, 20 mM NaF, 5 mM EDTA) with a cocktail of protease inhibitors (1 mM phenylmethylsulfonyl and 1 mg/ml each of pepstatin, leupeptin, N-tosyl-L-phenylalanyl chloromethyl ketone, N-tosyl-lysine chloromethyl ketone and soybean trypsin inhibitor). The tissue homogenates then underwent sedimentation at 100,000 × g for 20 mins and the supernatants were removed and kept as the HS fraction. Pellets were resuspended in 3 volumes per gram of tissue with HS buffer with 1 % Triton X-100 (HS/T buffer) and centrifuged at 100,000 × g for 20 mins. The supernatants were removed and kept as the HS/T fraction. The pellets were then homogenized in 3 volumes per gram of tissue with HS buffer with 1 M sucrose and centrifuged at 100,000 × g for 20 mins to float the myelin, which was discarded. Pellets were homogenized in 2 volumes per gram of tissue with radioimmunoprecipitation assay (RIPA) buffer (50 mM Tris, pH 8.0, 150 mM NaCl, 5 mM EDTA, 1 % NP-40, 0.5 % sodium deoxycholate, 0.1 % SDS) plus protease inhibitors and centrifuged at 100,000 × g for 20 mins. Supernatants were removed and kept as the RIPA fraction. Pellets were then homogenized in 1 volume per gram of tissue with 2 % SDS/4 M urea by probe sonication and kept as the SDS/U fractions. Protein concentrations of all fractions were determined by BCA assay using BSA (Pierce, Rockford, IL) as a standard. SDS sample buffer was added to the fractions which were incubated for 10 mins at 100 °C (HS and HS/T fractions) or at room temperature (SDS/U fraction only). Equal amounts of protein (10 μg for HS and HS/T, and 5 μg for SDS/U fractions) were resolved by SDS-PAGE and analyzed by immunoblot.

### Preparation of total protein lysate from mouse nervous tissue

Mice were humanely euthanized and the brain and spinal cord were harvested. The cortex and brainstem/spinal cord were dissected, placed in separate tubes and lysed with 2 % SDS/50 mM Tris, pH 7.5. Samples were sonicated using a probe sonicator until homogenous and incubated for 10 mins at 100 °C. Protein concentrations were determined by BCA assay using BSA as a standard. SDS sample buffer was added and equal amounts of protein (5 μg) were resolved by SDS-PAGE and analyzed by immunoblot.

### Immunoblotting analyses

Protein samples were resolved by electrophoresis on 8, 10 or 13 % polyacrylamide gels as indicated, then electrophoretically transferred to nitrocellulose membranes. Membranes were blocked with 5 % milk/Tris-buffered saline (TBS) then incubated overnight at 4 °C with primary antibodies diluted in 5 % milk/TBS (non-phospho antibodies) or 5 % BSA/TBS (phospho antibodies). Following washing, blots were incubated with HRP conjugated goat anti-mouse or donkey anti-rabbit secondary antibodies (Jackson Immuno Research Labs, West Grove, PA) diluted in 5 % milk/TBS for 1 h. Following washing, protein bands were visualized using Western Lightning-Plus ECL reagents (PerkinElmer, Waltham, MA) and images were captured using the GeneGnome XRQ system and GeneTools software (Syngene, Frederick, MD).

### Primary neuronal-glial cultures

Primary cultures (embryonic) were prepared from E16-E18 C3HBL/6 or αS null mouse brains. Cerebral cortices were dissected from E16-E18 mouse brains and were dissociated in 2 mg/ml papain (Worthington, Lakewood, NJ) and 50 μg/ml DNAase I (Sigma Aldrich, St. Louis, MO) in sterile HBSS at 37 °C for 20 mins. They were washed three times in sterile HBSS to inactivate the papain and switched to 1 % FBS in Neurobasal-A growth media (Gibco, Waltham, MA), which includes 1 % GlutaMax Supplement (Life Technologies, Carlsbad, CA), B-27 supplement, 100 U/ml penicillin and 100 μg/ml streptomycin. The tissue mixture was triturated three times using a 5 ml pipette followed by a Pasteur pipette, and strained through a 70 μm nylon cell strainer. The cell mixture was then centrifuged at 1300 × g for 3 mins, and resuspended in fresh Neurobasal-A media. They were then plated on Nunc Lab-Tek II CC2 chamber slides (Life Technologies, Carlsbad, CA) at around 100,000-200,000 cells/cm^2^. Cells were maintained in the Neurobasal-A growth media without FBS at 37 °C in a humidified 5 % CO_2_ chamber. The cultures were maintained for 6 days followed by another 8 days without or with 20 μg/ml water bath sonicated mouse αS fibrils prepared as previously described [34, 35].

### Immunofluorescence microscopy analysis

For double immunofluorescence analysis, cells were washed with PBS and fixed with 4 % paraformaldehyde/PBS. Following PBS washes, cells were blocked with 5 % FBS/PBS/0.1 % Triton X-100 for 30 mins. Cultures were stained with primary antibodies followed by Alexa-fluor 488 and 594 conjugated secondary antibodies (Invitrogen, Carlsbad, CA). Nuclei were counterstained with 4′,6-diamidino-2-phenylindole (DAPI; Invitrogen, Carlsbad, CA), and coverslips were mounted using Fluoromount-G (Southern Biotech, Birmingham, AL). Immunofluorescence images were captured with an Olympus BX51 fluorescence microscope mounted with a DP71 digital camera (Olympus, Center Valley, PA).

## Results

### Assessment of NFL and αS phosphorylation by casein kinase II and polo-like kinases

**Fig. 1:**
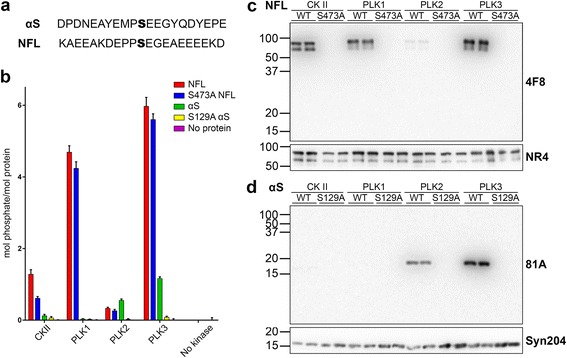
In vitro phosphorylation of NFL and αS by CKII and PLK1, 2 and 3. **a** Amino acid sequences surrounding the target phosphorylation sites (bold) of Ser129 for αS, and Ser473 for NFL. **b** Quantitative analysis of the stoichiometric phosphorylation of recombinant αS, S129A αS, NFL and S473A NFL in in vitro kinase reactions as described in “Materials and methods” with CKII, PLK1, PLK2 or PLK3. All reactions were performed in triplicate. **c** Immunoblotting analyses of NFL and S473A NFL used in in vitro kinase reactions with CKII, PLK1, PLK2 or PLK3. Proteins were resolved onto 8 % polyacrylamide gel and probed with 4F8 (pSer473 NFL) and NR4 (anti-NFL) antibodies. **d** Immunoblotting analyses of αS and S129A αS used in in vitro kinase reactions with CKII, PLK1, PLK2 or PLK3. Proteins were resolved onto 13 % polyacrylamide gels and probed with 81A (pSer129 αS) and Syn 204 (anti-αS). The mobility of molecular mass markers are shown on the left

CKII was previously reported as a major kinase responsible for the phosphorylation of αS, more specifically at Ser129 [19, 36, 37], and NFL [38–40]. Characterization of NFL phosphorylation by CKII was predominantly focused on Ser473, which is the major phosphorylation site in NFL in vivo [40, 41]. Phosphorylation of αS at Ser129 can be mediated by many additional kinases [19, 42–47] but some PLKs appear to be quite efficient in driving this modification [42, 43, 47]. To compare the relative abilities of CKII and PLK 1, 2 and 3 to phosphorylate αS and NFL, and use this information to generate phosphorylated protein that can be used to assess the specificity of novel antibodies (see below), we assessed the relative but quantitative in vitro phosphorylation of αS and NFL with CKII and PLK 1, 2 and 3 (Fig. 1b). In these parallel kinase conditions, PLK3 was the most efficient at phosphorylating αS at a stoichiometric ratio of close to 1 mol of phosphate per 1 mol of protein. This phosphorylation was almost completely abolished for S129A αS indicating that Ser129 is the major αS phosphorylation site for PLK3. NFL was phosphorylated by PLK1 and PLK3 at multiple amino residues as shown by the greater stoichiometric molecular ratio of approximately 4.7 and 6, respectively. In addition, the presence of the S473A mutation in NFL only reduced the amount of phosphate incorporation by PLK1 and PLK3 by 0.45 and 0.37 mol phosphate/mol protein respectively, indicating that Ser473 is not the major site targeted by these kinases. CKII phosphorylated NFL at a ratio of 1.28 mol phosphate/mol protein, and this was reduced to 0.61 for S473A NFL indicating that, while this is not the only site, it is the major target site for this kinase.

### Generation of novel pSer473 NFL and pSer129 αS antibodies

To provide more reagents to study the contribution of aberrant accumulation and/or distributions of αS and NFL in neurodegenerative diseases, we generated novel monoclonal antibodies against the pSer473 epitope in NFL and the pSer129 epitope in αS. For the pSer473 NFL epitope we used a synthetic peptide with phosphorylated serine and the adjacent 7 amino acids residues on either side in the human NFL sequence (Table 1). We identified and characterized (see below) one clone termed 4F8 that was relatively specific for pSer473 in NFL.

For the pSer129 αS epitope we performed multiple attempts to produce αS antibodies that were 1) phospho-specific and 2) relatively specific in detecting Lewy pathology. For our most successful approach, the mice were first immunized with the pSer129long peptide (αS residues 121–137) then 3 weeks later with the pSer129short peptide (αS residues 124–134; Table 1). The final IP injection used a 1:1 ratio of the 2 peptides and the initial ELISA antibody screen used the pSer129long peptide. We immunized several mice using this strategy and kept the most promising hybridomas from our initial ELISA and IHC screens for further characterization. We have denoted these antibodies LS, meaning long then short to indicate the peptides that were used for immunization.

### Characterization of novel monoclonal antibody specificities using phosphorylated recombinant proteins

To confirm our findings from the in vitro kinase reactions and initially characterize the specificity of the 4F8 antibody, we conducted immunoblotting analysis with NFL and S473A NFL individually phosphorylated with CKII and PLK1, 2 and 3 (Fig. 1c). As expected, the signal was the weakest with PLK2 and the signal was abolished for S473A NFL phosphorylated with any of these kinases indicating that 4F8 is specific for NFL phosphorylated at Ser473. Immunoblotting with the previously generated pSer129 αS antibody 81A [19] was consistent with the in vitro radioactivity kinase studies in terms of relative phosphorylation of αS by these kinases (Fig. 1d). These results show that PLK3 strongly phosphorylates both NFL at Ser473 and αS at Ser129, and that CKII is the preferred kinase for NFL at this site. Therefore we chose to use these kinase reactions to further screen the specificity of our novel antibodies.

**Fig. 2:**
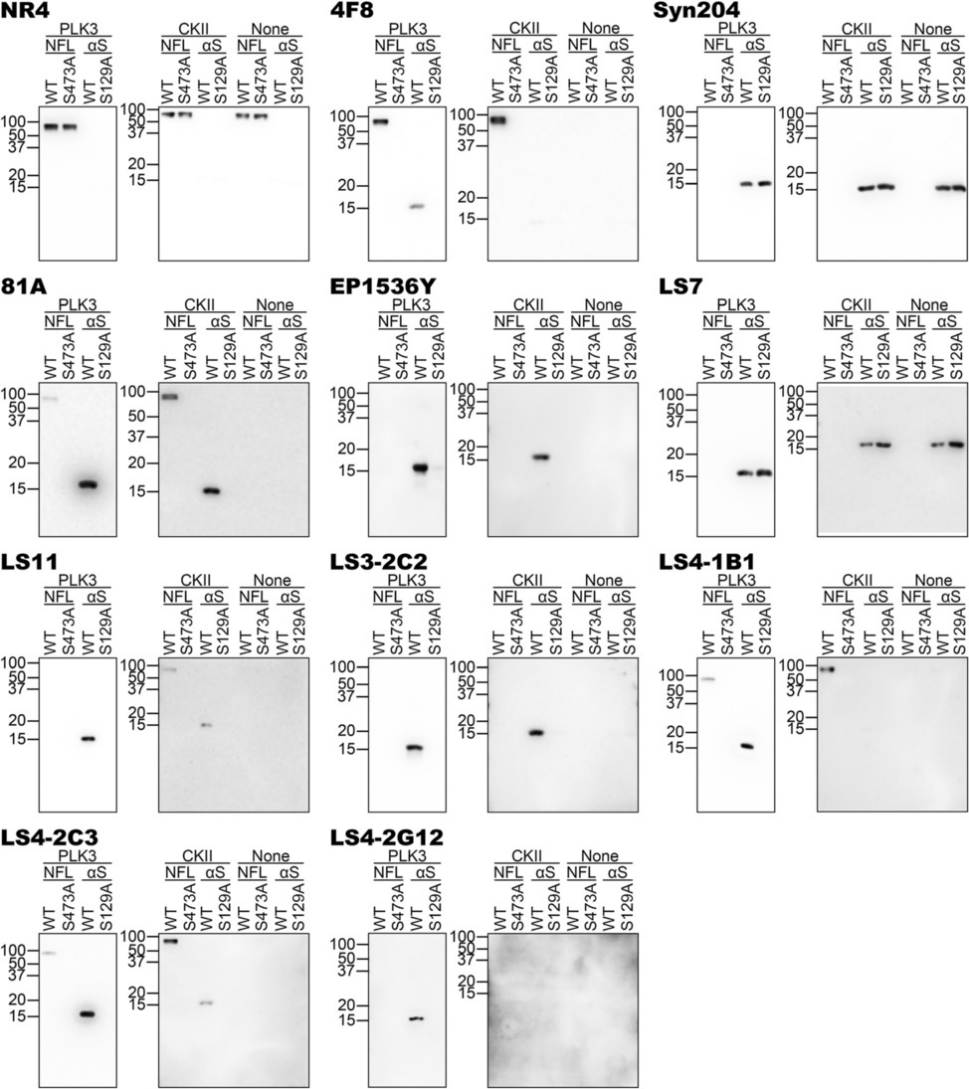
Specificity of novel pSer129 αS antibodies as determined by immunoblotting with NFL and αS proteins phosphorylated in vitro with PLK3 or CKII. NFL, S473A NFL, αS and S129A αS were incubated with PLK3 or CKII. The proteins were resolved onto 13 % polyacrylamide gels and analyzed by immunoblotting with novel αS antibodies (as indicated above each blot) LS7, LS11, LC2-2C2, LS4-1B1, LS4-2C3 and LS4-2G12 and novel NFL antibody 4F8, to demonstrate their specificity. NR4 (anti-NFL) and Syn 204 (anti-αS) were included to show each protein, respectively. Previously reported pSer129 αS antibodies 81A and EP1536Y were included for comparison. Reactions were performed in duplicate. Antibody 4F8, generated against the pSer473 NFL epitope, can cross react with αS phosphorylated at Ser129. Antibodies 81A, LS11, LS4-1B1 and LS4-2C3 generated against the pSer129 αS epitope can cross react with NFL phosphorylated at Ser473. Antibodies EP1536Y, LS3-2C2 and LS4-2G12 generated against the pSer129 αS epitope reacted only with phosphorylated WT αS. Antibody LS7 generated against the pSer129 αS epitope detected both phospho- and non-phospho-αS. The mobility of molecular mass markers are shown on the left

We performed immunoblot analyses of CKII and PLK3 kinase reactions with WT and S473A NFL, and WT and S129A αS (Fig. 2). Immunoblots with anti-NFL antibody NR4 and anti-αS antibody Syn 204 are included to demonstrate the respective proteins. In the immunoblots loaded with CKII reactions, 4F8 appeared specific for phosphorylated NFL, however the blots with the PLK3 reactions showed that it can also detect αS phosphorylated at Ser129. As previously published, 81A also cross reacted with NFL phosphorylated at Ser473 [18], particularly when phosphorylated by CKII. LS3-2C2 and the commercially available EP1536Y were specific for αS phosphorylated at Ser129. LS7 reacted with αS independent of its phosphorylation state. Antibodies LS11, LS4-1B1 and LS4-2C3 were specific for αS phosphorylated at Ser129, however they could also react to some extent with NFL phosphorylated at Ser473. LS4-2G12 only showed reactivity with αS phosphorylated at Ser129 by PLK3, but it did not react with αS modified with CKII due to its low level of phosphorylation, suggesting that it is a weaker antibody.

### Assessment of antibody specificity using biochemically fractionated mouse nervous tissue

**Fig. 3:**
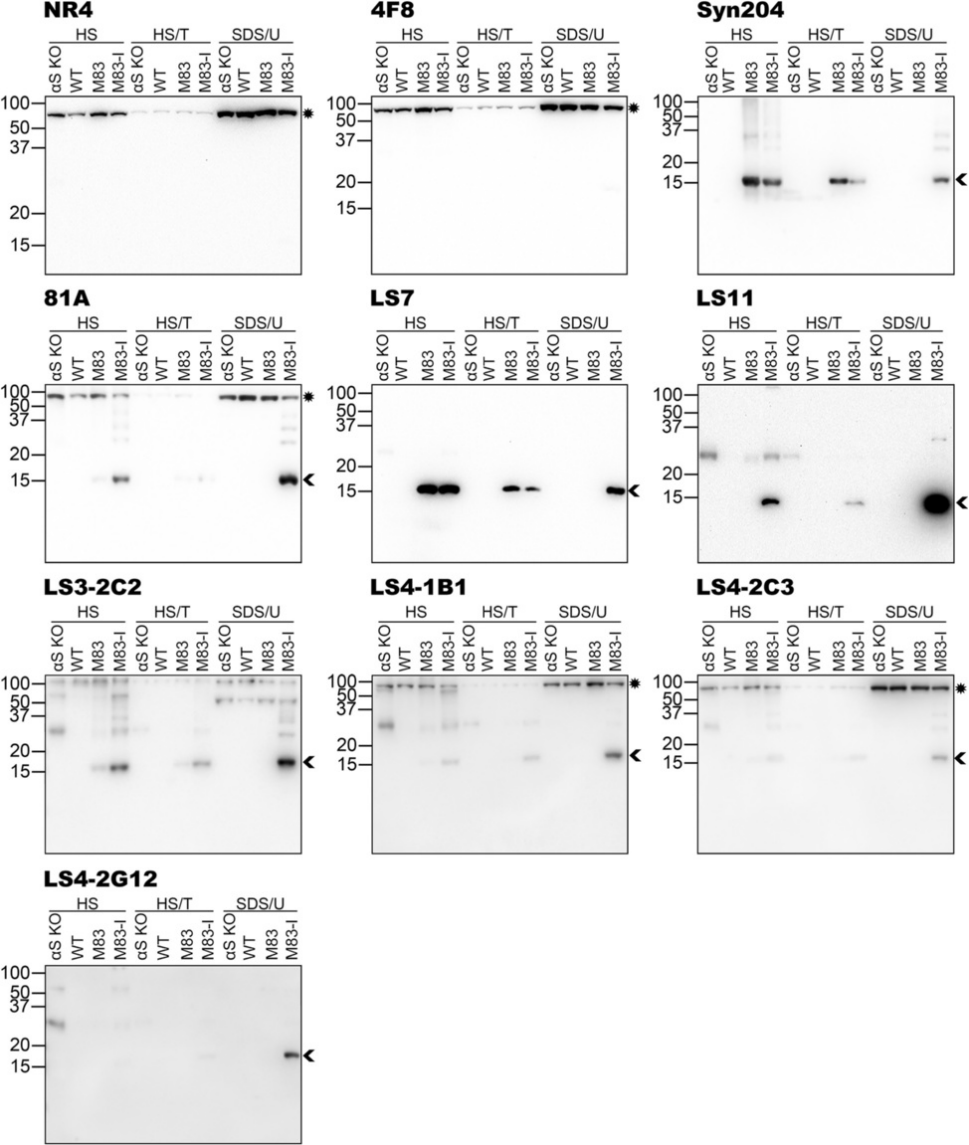
Characterization of the specificity of novel pSer129 αS antibodies by immunoblotting analyses using biochemically fractionated brainstem/spinal cord mouse tissues. Brainstem/spinal cord from an αS null (αS KO), a WT, a 2 month old non-symptomatic M83 (M83) and a 12 month old motor impaired M83 (M83-I) mouse were biochemically fractionated into high salt (HS), high salt/Triton X-100 (HS/T) and SDS/urea (SDS/U) fractions as described in “Materials and methods”. 10 μg of HS and HS/T, and 5 μg of SDS/U fractions were resolved onto 13 % polyacrylamide gels and analyzed by immunoblotting with each antibody indicated above. The protein band identified by an arrowhead is αS, while an asterisk depicts NFL. The mobility of molecular mass markers are shown on the left

**Fig. 4:**
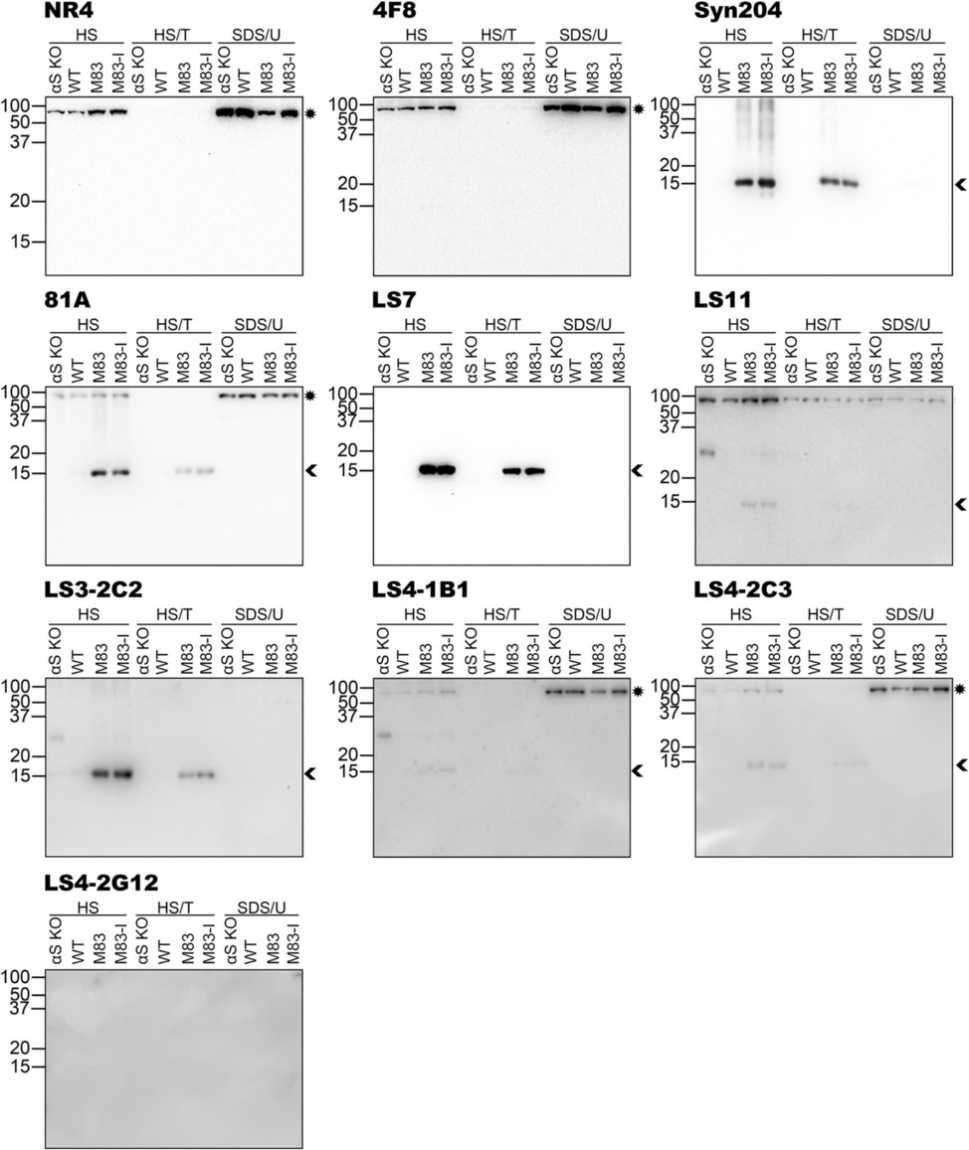
Characterization of the specificity of novel pSer129 αS antibodies by immunoblotting analyses using biochemically fractionated cerebral cortex mouse tissues. Cerebral cortex from an αS null (αS KO), a WT, a 2 month old non-symptomatic M83 (M83) and a 12 month old motor impaired M83 (M83-I) mouse were biochemically fractionated into high salt (HS), high salt/Triton X-100 (HS/T) and SDS/urea (SDS/U) fractions as described in “Materials and methods”. 10 μg of HS and HS/T and 5 μg of SDS/U fractions were resolved onto 13 % polyacrylamide gels and analyzed by immunoblotting with each antibody indicated above. The protein band identified by an arrowhead is αS, while an asterisk depicts NFL. The mobility of molecular mass markers are shown on the left

**Fig. 5:**
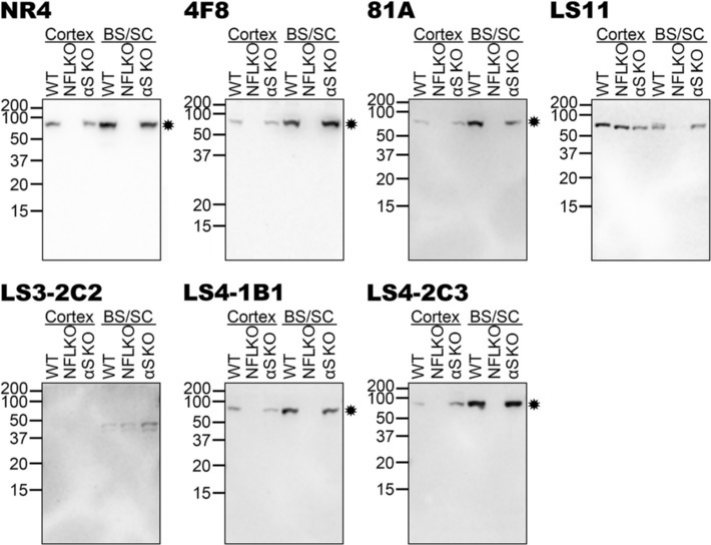
Characterization of the specificity of novel pSer129 αS antibodies by immunoblotting analyses using total lysates of cortex and brainstem/spinal cord tissues from WT and NFL null mice. Cerebral cortex and brainstem/spinal cord (BS/SC) from a WT, an NFL null (NFL KO) and an αS null (αS KO) mouse were dissected and lysed in 2 % SDS/ 50 mM Tris pH 7.5 as described in “Materials and methods”. Equal amounts of proteins (5 μg) from each sample was resolved onto 10 % polyacrylamide gels and analyzed by immunoblotting with each antibody indicated above. The protein band identified by an asterisk depicts NFL. The mobility of molecular mass markers are shown on the left

To assess for the more global specificity of the new antibodies, we performed immunoblot analyses of sequentially fractionated mouse brainstem/spinal cord (Fig. 3) and cerebral cortex (Fig. 4) tissue from αS null (αS KO), WT, 2 month old non-symptomatic M83 (M83) and 12 month old motor-impaired M83 (M83-I) mice. The M83-I mouse contains pathological αS aggregates predominantly in the brainstem/spinal cord [25]. Immunoblots with anti-NFL antibody NR4 and anti-human αS antibody Syn 204 are included to demonstrate the presence and distribution of these respective proteins. In these analyses, 4F8 was quite specific, detecting predominantly NFL and only a very faint phospho-αS band in the brainstem/spinal cord SDS/U fraction of the M83-I mouse (Fig. 3). LS7 showed a similar pattern of reactivity to Syn 204, again indicating that it is not phospho-specific (Figs. 3 and 4). Antibodies LS4-1B1, LS4-2C3 and 81A could all react with pathological αS that accumulates in the brainstem/spinal cord SDS/U fraction of M83-I mice (Fig. 3), but they also cross reacted to some extent with phosphorylated NFL present in the brainstem/spinal cord and cortex of all these mice (Figs. 3 and 4). This cross-reactivity to NFL was confirmed by immunoblotting of total brainstem/spinal cord and cortex tissues from WT, NFL null and αS null mice (Fig. 5). LS11 detected aggregated phosphorylated αS (Fig. 3) but also cross-reacted with NFL, as shown by the reduction in signal of the ~70 kDa band in the total brainstem/spinal cord extract from the NFL null mice (Fig. 5). However, it also reacted with another protein of approximately the same size as NFL prevalent in the cortex, even in NFL and αS null mice (Fig. 5). This protein also has a different biochemical fractionation profile compared to NFL in the cortex (Fig. 4). Antibodies LS11, LS3-2C2, LS4-1B1, LS4-2C3 and LS4-2G12 also detected a ~30 kDa non-αS protein. LS3-2C2 weakly labeled a few additional protein bands in the brainstem/spinal cord lysates of all mice indicating that these are not αS. Overall, LS4-2G12 appeared to be the most specific new phospho-αS antibody.

### IHC analyses of mouse and human nervous tissue with new pSer473 NFL and pSer129 αS antibodies

**Fig. 6:**
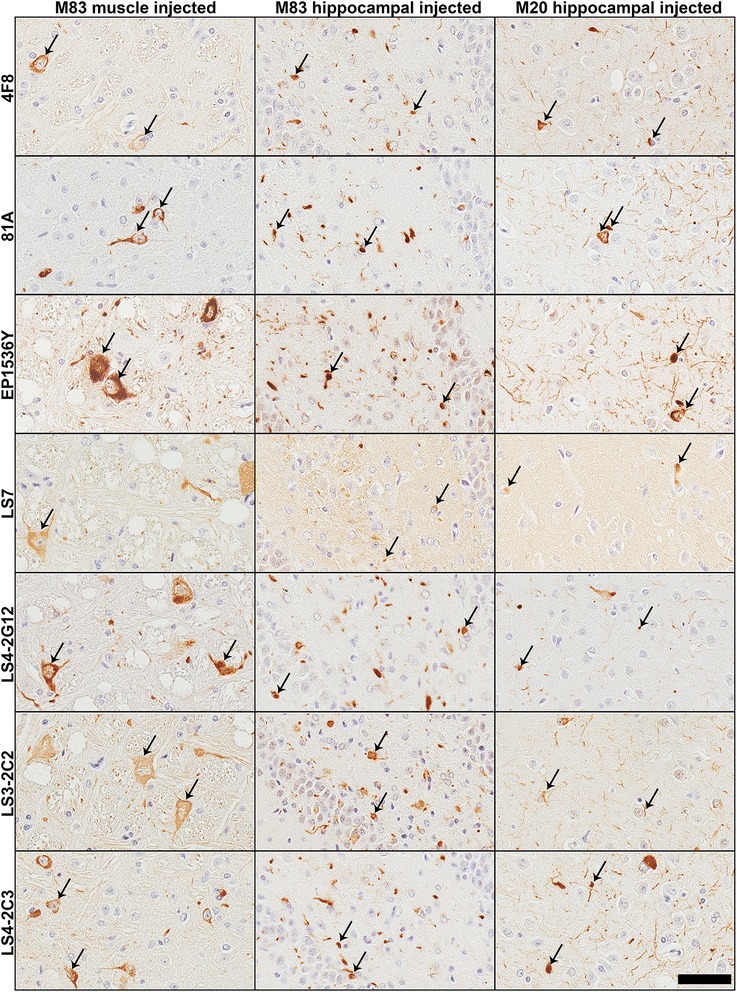
Comparison of novel antibodies in detecting pathological inclusions in αS transgenic mice injected with αS fibrils in the periphery (intramuscular) or the brain (hippocampus). Representative images of IHC staining of tissue from M83 αS transgenic mice injected in the gastrocnemius muscle, and M83 and M20 αS transgenic mice injected in the hippocampus with recombinant preformed αS fibrils. Images were taken from the brainstem (muscle injection) and the hippocampus (hippocampal injection). Antibodies EP1536Y and 81A showed robust staining of induced inclusions (arrows). LS7 stained inclusions weakly with higher general labeling. Novel antibodies raised against the pSer129 αS epitope LS4-2G12, LS3-2C2 and LS4-2C3 or to the p473 NFL epitope all stained the induced inclusions in these models. Scale bar = 50 μm

**Fig. 7:**
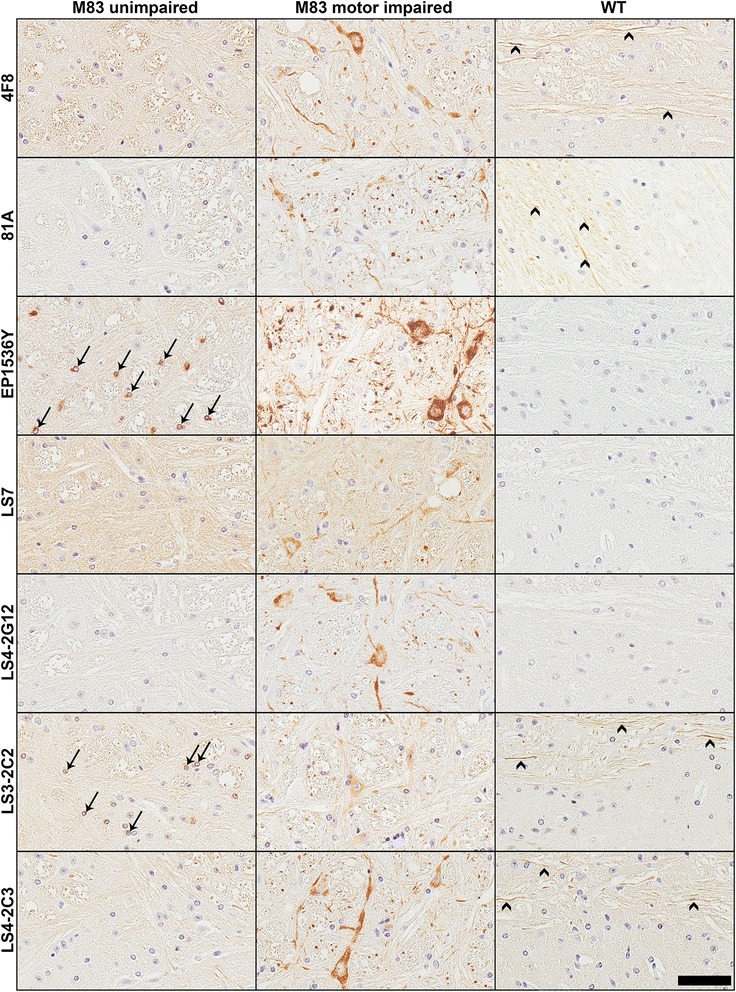
Comparison of pSer129 antibody IHC staining using naïve αS transgenic and WT mice. Representative images of IHC staining of brainstem tissue from a 7 month old non-symptomatic homozygous M83 mouse (M83 unimpaired), a 12 month old motor impaired homozygous M83 mouse (M83 motor impaired) and a WT mouse. All antibodies stained perikaryal and neuritic inclusions in the M83 diseased mouse. LS7 showed weaker reactivity to αS pathology than the other antibodies and showed stronger diffused signal in the αS transgenic mice compared to the WT mouse. LS3-2C2 also showed weak staining of pathology. In addition to the pathology in motor impaired M83 mouse, antibodies 4F8, LS3-2C2, LS4-2C3 and 81A also labeled neuronal projections even in WT mice (arrowheads). EP1536Y exhibited some nuclear staining in some mouse sections (arrows), as did LS3-2C2, but much weaker than EP1536Y (eg. see arrows in the M83 unimpaired mouse). Scale bar = 50 μm

Using our new and previously generated pSer129 αS antibodies and the new pSer473 NFL antibody 4F8, we performed IHC analyses on a cohort of mouse tissue derived from αS transgenic and WT mice (Table 2) and human autopsy cases with a diagnosis of PD, DLB or MSA (Table 3). Our mouse tissue cohort, included previously described [26–28] tissue of αS transgenic mice injected with αS fibrils either in the gastrocnemius muscle (M83 line) or hippocampus that induces the formation of αS inclusion pathology (M83 and M20 lines; Fig. 6), and naïve αS transgenic and WT mice (Fig. 7). The tissue in this cohort was fixed with 150 mM NaCl/70 % ethanol. The staining revealed that all of the anti-αS antibodies tested were able to reliably stain αS inclusion pathology in this cohort. Likewise, 4F8 stained many inclusions. EP1536Y also stained inclusions, however in some sections it had a tendency to non-specifically react with cellular nuclei (see Fig. 7). Phosphorylation-independent antibody, LS7 stained inclusions, but weaker than the phospho-specific antibodies. Interestingly, the general baseline staining of LS7 was only evident in the αS transgenic and not in the WT mice (Fig. 7), because it is specific for human αS (Additional file 1: Figure S1). Antibodies that could cross-react with phospho-NFL such as LS4-2C3, as well as LS3-2C2 which does not detect phospho-NFL, also demonstrated staining of axonal processes similar to 4F8 (Fig. 7).

**Fig. 8:**
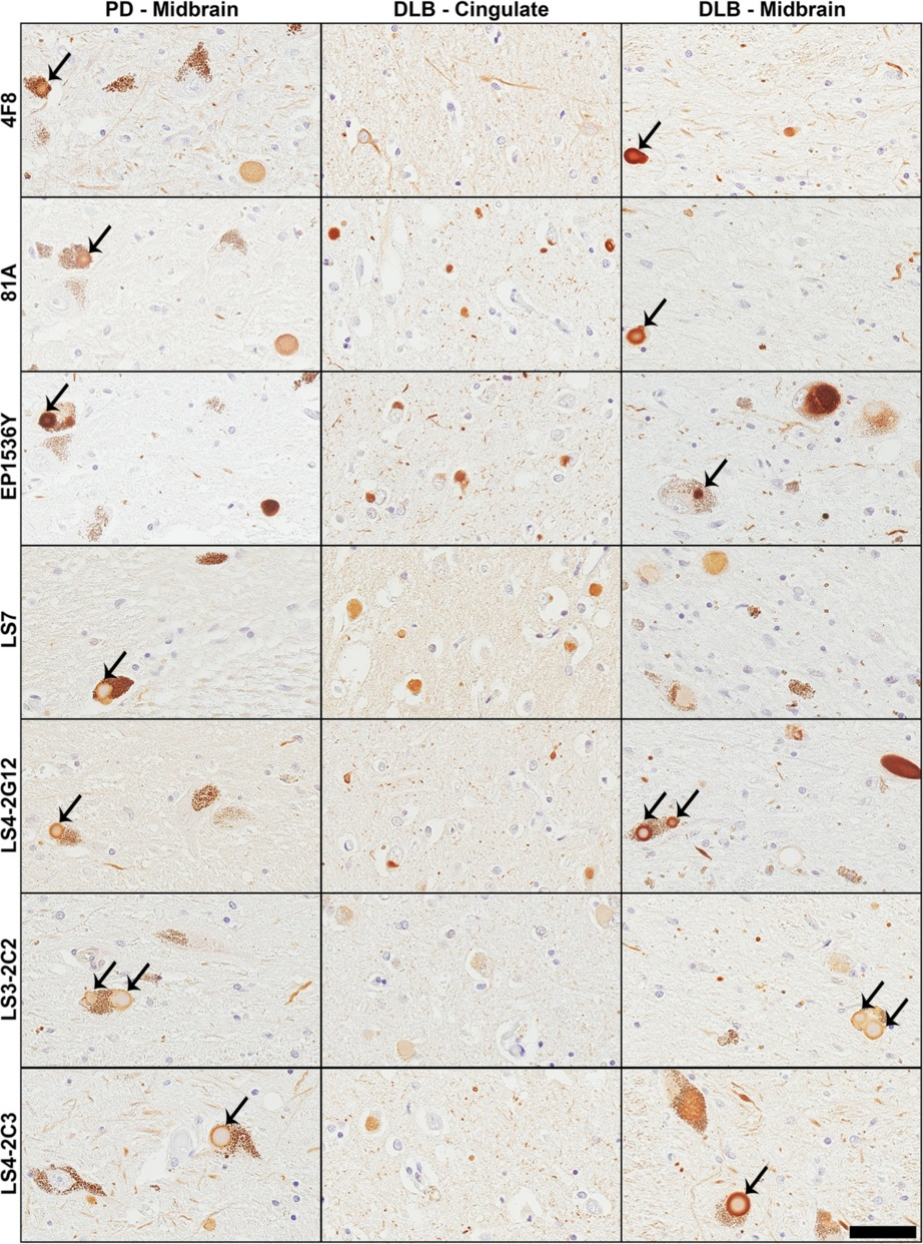
IHC analyses of brain sections of individuals with Parkinson’s disease and dementia with Lewy bodies. Representative IHC staining of brain tissue sections from PD and DLB patients. Images were taken from the midbrain (PD and DLB) or cingulate cortex (DLB only). All of the antibodies stained LBs within dopaminergic neurons in the midbrain sections for both PD and DLB. Arrows show LBs in some examples within cells containing neuromelanin. Scale bar = 50 μm

**Fig. 9:**
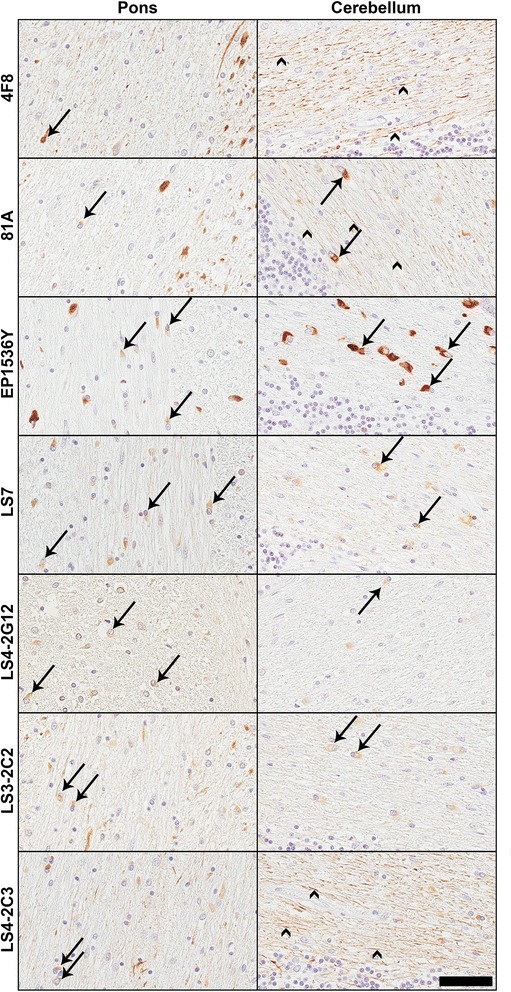
IHC analyses of brain sections of individuals with multiple system atrophy. Representative IHC staining of brain tissue sections from MSA patients. Images were taken from the pons or cerebellum. Arrows show GCIs. All of the antibodies stained inclusions, to some extent. EP1536Y showed the strongest staining. 4F8 stained only rare inclusions, but abundantly labeled axons (arrowheads). Antibodies 81A and LS4-2C3 also displayed strong reactivity to axons in the cerebellum. Antibodies LS4-2G12 and LS3-2C2 displayed weaker staining of inclusions in the cerebellum. Scale bar = 50 μm

For our human autopsy cohort, we performed IHC on sections of midbrain (PD and DLB), cingulate cortex (DLB; Fig. 8), and pons and cerebellum (MSA; Fig. 9). All of the antibodies tested showed robust reactivity to the αS inclusion pathology in the PD and DLB cases, apart from LS3-2C2 which displayed weaker staining particularly in the DLB cingulate cortex (Fig. 8). All pSer129 αS antibodies stained GCIs in the MSA tissue; however the strength of the signal differed extensively between the antibodies (Fig. 9). For example, EP1536Y showed the strongest staining, while LS4-2G12 and LS3-2C2 showed weaker staining, especially in the cerebellum. Although LS4-2C3 labeled αS inclusions well in the pons, its reactivity with NFL made it difficult to make out many inclusions within the cerebellum; the axonal staining being on par with that of 4F8. 4F8 itself was able to stain some GCIs, however these were rare, and whether this is really pSer473 NFL staining, or just cross-reactivity with pSer129 αS remains in question.

Using mouse tissue fixed using formalin or ethanol (150 mM NaCl/70 % ethanol), we confirmed that all the antibodies could be used to stain tissue preserved with both types of fixatives (Additional file 2: Figure S2).

### Immunofluorescence analyses of αS aggregation in primary neuronal-glial cultures

**Fig. 10:**
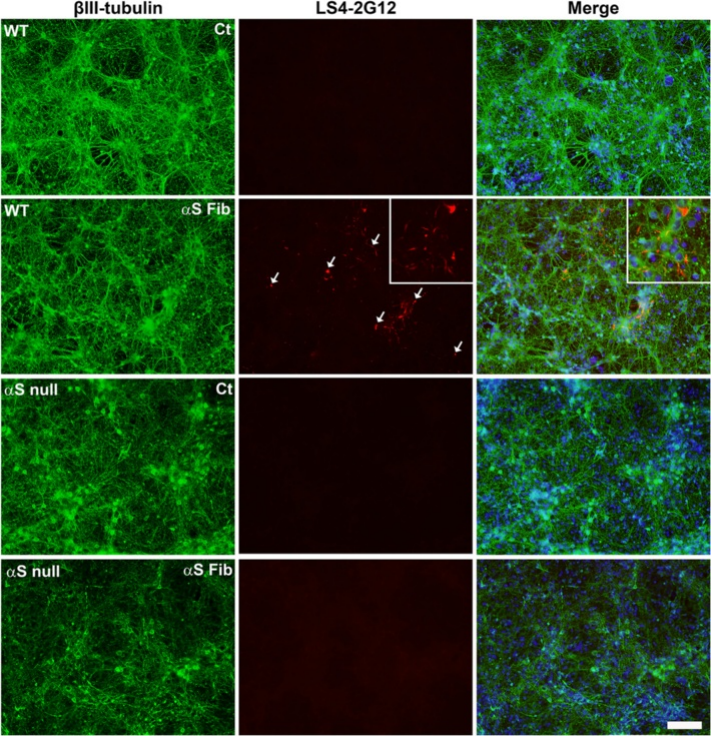
Analysis of the induction of endogenous αS aggregation by treatment with exogenous αS mouse fibrils in primary neuronal-glial cultures using antibody LS4-2G12. Primary neuronal-glial cultures from WT mice or αS null mice were cultured for 6 days and either maintained without other treatment for 8 days (Ct) or treated with mouse αS fibrils (20 μg/ml; αS Fib) for 8 days. Double immunofluorescence analysis with antibodies LS4-2G12 (red) and specific neuronal marker βIII-tubulin (green) was performed. Cells were also counterstained with DAPI and merged images are shown. Higher magnification LS4-2G12 and merged images are shown on the far right. Arrows depict induced labeled αS aggregates. Scale bar = 100 μm and 250 μm for the higher magnification images on the right

Using only the new pSer129 αS antibodies that do not cross-react with phospho-NFL, we determined if these could be used to monitor the aggregation of αS in primary neuronal-glial cultures induced by the addition of exogenous αS preformed fibrils. LS4-2G12 readily detected neuronal phosphorylated αS aggregates, and the paucity of these inclusions in cultures from αS null mice confirmed that these were comprised of endogenous αS (Fig. 10). Antibody LS3-2C2 could also detect inclusions comprised of endogenous αS in primary neuronal-glial cultures treated with exogenous αS fibrils, but this antibody was less useful due to the non-specific cross-reactivity with cellular nuclei (Additional file 3: Figure S3).

## Discussion

In this study we addressed the need for specific, well characterized antibodies to pathological αS by generating monoclonal antibodies against the pSer129 αS epitope, as most existing pSer129 αS antibodies have been reported to cross react with additional proteins [4, 18]. For example, we previously showed that pSer129 antibody, 81A cross-reacts with pSer473 NFL [18] while antibody MJF-R13(8–8) that does not detect phosphorylated NFL, is still highly non-specific and cannot be used to assess αS pathology [4]. The difficulty of making antibodies specific to pSer129 αS is not entirely surprising. This site can be phosphorylated in vitro by multiple serine/threonine kinases, including CKI and II, PLK 1, 2 and 3, and G-protein coupled receptor kinases (GRKs) [19, 37, 42–45, 47]. Each of these kinases in turn can phosphorylate numerous protein targets, which contain a certain level of homology in the amino acid sequence surrounding each phosphorylation site. As such, we show that both αS and NFL can be phosphorylated by CKII, PLK2 and PLK3, but Ser129 in αS is a preferential site for PLK2 and PLK3, demonstrating higher phosphorylation efficiency. Comparatively, CKII preferentially phosphorylates Ser473 in NFL and this site can be phosphorylated by PLK1, 2 and 3, but these enzymes prefer to modify other sites within NFL.

The pSer473 NFL epitope contains some sequence identity to pSer129 αS (Fig. 1a), but the fact that a disproportionate number of antibodies to pSer129 αS react with pSer473 NFL indicates that these two epitopes share additional and specific conformational cues beyond simply the primary sequence. This notion is further demonstrated by the findings that antibody 4F8 generated against the pSer473 NFL epitope can cross-react, to some extent, with pSer129 in αS. In addition, most of the pSer129 αS antibodies that react with pSer473 NFL are relatively specific for only these 2 epitopes, even on immunoblot analysis of whole brain and spinal cord lysates, and they do not react with other phospho-epitopes within NFL that can be phosphorylated by PLKs. The complications in using pSer129 αS antibodies that are confounded by pSer473 NFL reactivity in staining tissues are compounded by 1) the high abundance of NFL in the nervous tissue, 2) that Ser473 in NFL is intrinsically highly phosphorylated [41], and 3) neurofilaments are natively assembled into fibrillar structures that can resemble αS polymerized to form pathological inclusions.

Our studies have yielded and characterized 2 new monoclonal antibodies, LS3-2C2 and LS4-2G12, in addition to the commercial antibody EP1536Y that does not cross-react with phosphorylated NFL. However, LS3-2C2 reacted with additional non-αS proteins and its tendency to non-specifically react with cellular nuclei renders it less useful for some studies. The issue of non-specific staining of cellular nuclei was intermittently observed with EP1536Y. In addition, pSer129 antibodies LS11, LS3-2C2, LS4-1B1, LS4-2C3 and LS4-2G12 also detect a ~30 kDa non-αS protein in mouse nervous tissue, which was most obvious in the αS null mouse. Nevertheless, pSer129 αS antibody LS4-2G12 was the most specific of the antibodies tested, but it did not stain pathological inclusions as strongly as other pSer129 αS antibodies, which could be due to lower affinity and/or cross-reactivity. These new pSer129 αS antibodies will be very useful tools, especially in the research community where they are commonly used for tracking the induction and spread of αS pathology in experimental models. The cross-reactivity of pSer129 αS antibodies is less of a concern in a clinical setting, where diagnosis of synucleinopathy is determined using a well-established set of non-phospho αS antibodies.

To our knowledge, antibody 4F8 is the first monoclonal pSer473 NFL antibody reported, although a polyclonal antibody to this epitope was reported in 1999 [40]. This antibody will be a very useful tool when used together with pSer129 αS antibodies that do not cross-react with phosphorylated NFL, to expand studies of aberrant phosphorylated neurofilaments within neuropathological inclusions. NFL is the core neurofilament subunit required for proper neurofilament assembly and neurofilaments are present in many different types of inclusions associated with neurodegenerative diseases [48], for example neurofilament inclusion disease (NFID), a rare, sporadic disease with features of frontotemporal dementia [49–53]. In addition, neuroaxonal spheroids comprised of bundles of neurofilaments are a common feature in patients with amyotrophic lateral sclerosis [54, 55] and neuroaxonal dystrophies [56–58]. Furthermore, neurofilaments typically accumulate within neurofibrillary tangles of Alzheimer’s disease [59, 60].

## Conclusions

In conclusion, even though pSer129 αS antibody staining can be a very sensitive tool for detecting aberrantly aggregated αS and pathological inclusions, specificity is a critical issue that needs to be considered for accurate assessments. The intrinsic similarities in primary sequence surrounding this site to other proteins that are phosphorylated by mutual kinases and the apparent additional structural homology to the pSer473 NFL epitope complicates the generation of highly specific antibodies. Nevertheless, from a series of new monoclonal antibodies, we were able to identify LS4-2G12 that is relatively specific and useful in detecting αS aggregates in human brains and experimental models. However, given the varied specificity and properties of pSer129 αS antibodies, as demonstrated here, it should be preferred as a common practice to use a combination of several pSer129 αS antibodies in addition to an αS antibody to accurately assess neuropathological changes in αS.

## Abbreviations

αS, α-synuclein; αS fib, α-synuclein fibrils; ATP, adenosine triphosphate; BCA, bicinchoninic acid; BSA, bovine serum albumin; BS/SC, brainstem/spinal cord; CK, casein kinase; Ct, control; DAB, 3, 3′diaminobenzidine; DAPI, 4′,6-diamidino-2-phenylindole; DLB, dementia with Lewy bodies; DMEM, Dulbecco’s Modified Eagle Medium; DTT, dithiothreitol; *E.coli, Escherichia coli*; EDTA, ethylenediaminetetraacetic acid; EGTA, ethylene glycol-bis(β-aminoethyl ether)-N,N,N’,N’-tetraacetic acid; ELISA, enzyme-linked immunosorbent assay; FBS, fetal bovine serum; GCI, glial cytoplasmic inclusion; GRK, G-protein coupled receptor kinase; HBSS, Hank’s balanced salt solution; HEPES, 4-(2-hydroxyethyl)-1-piperazineethanesulfonic acid; HRP, horse radish peroxidase; HS, high salt; HS/T, high salt/Triton X-100; IHC, immunohistochemistry; IP, intraperitoneal; KO, null (knock-out); LB, Lewy body; M83-I, motor impaired M83 mouse; MSA, multiple system atrophy; NCI, neuronal cytoplasmic inclusion; NFID, neurofilament inclusion disease; NFL, low molecular mass neurofilament subunit; PAGE, polyacrylamide gel electrophoresis; PBS, phosphate buffered saline; PD, Parkinson’s disease; PEG, polyethylene glycol; PLK, polo-like kinase; RIPA, radioimmunoprecipitation assay; pSer, phosphorylated serine; SDS, sodium dodecyl sulfate; SDS/U, sodim dodecyl sulfate/urea; TBS, Tris-buffered saline; WT, wild-type

## Additional files

Additional file 1: Figure S1. Specificity of novel antibody LS7 for human αS. (**a**) Recombinant human and mouse αS (50 ng) were resolved onto 13 % polyacrylamide gels and analyzed by immunoblotting with LS7 and anti-αS antibody SNL-4 (residues 2–12 of αS). The mobility of molecular mass markers are shown on the left. (**b**) Sequence of the pSer129long peptide (human αS) with the corresponding mouse αS sequence underneath. The line indicates amino acids that are not shared. (TIF 216 kb) Additional file 2: Figure S2. IHC analyses showing antibody reactivity in formalin and ethanol fixed tissues. Representative IHC staining of the brainstem of M83 mice injected in the gastrocnemius muscle with αS fibrils. The tissue of one mouse (*left*) was fixed in 150 mM NaCl/70 % ethanol and the other (*right*) was fixed in formalin. All of the antibodies were able to stain pathology in both formalin and ethanol fixed tissue, however some staining appeared weaker in the formalin fixed tissue (LS4-2G12 and LS3-2C2). Scale bar = 50 μm. (TIF 9583 kb) Additional file 3: Figure S3. Analysis of the induction of endogenous αS aggregates with exogenous αS mouse fibrils in primary neuronal-glial cultures using antibody LS3-2C2. Primary neuronal-glial cultures from WT mice or αS null mice were cultured for 6 days and either maintained without other treatment for 8 days (Ct) or treated with mouse αS fibrils (20 μg/ml; αS fib) for 8 days. Double immunofluorescence analysis with antibodies LS3-2C2 (*red*) and specific neuronal marker βIII-tubulin (*green*) was performed. Cells were also counterstained with DAPI and merged images are shown. Higher magnification merged images are shown on the far right. *Arrows* depict induced labeled αS aggregates and arrowheads depict nuclear staining. Bar = 100 μm and 250 μm for the higher magnification images on the right. (TIF 7508 kb)

## References

[1] Goedert M (2001). Alpha-synuclein and neurodegenerative diseases. Nat Rev Neurosci.

[2] Goedert M, Spillantini MG, Del Tredici K, Braak H (2013). 100 years of Lewy pathology. Nat Rev Neurol.

[3] Spillantini MG, Schmidt ML, Lee VM, Trojanowski JQ, Jakes R, Goedert M (1997). Alpha-synuclein in Lewy bodies. Nature.

[4] Uchihara T, Giasson BI (2016). Propagation of alpha-synuclein pathology: hypotheses, discoveries, and yet unresolved questions from experimental and human brain studies. Acta Neuropathol.

[5] Spillantini MG, Crowther RA, Jakes R, Cairns NJ, Lantos PL, Goedert M (1998). Filamentous alpha-synuclein inclusions link multiple system atrophy with Parkinson’s disease and dementia with Lewy bodies. Neurosci Lett.

[6] Tu PH, Galvin JE, Baba M, Giasson B, Tomita T, Leight S (1998). Glial cytoplasmic inclusions in white matter oligodendrocytes of multiple system atrophy brains contain insoluble alpha-synuclein. Ann Neurol.

[7] Hamilton RL (2000). Lewy bodies in Alzheimer’s disease: a neuropathological review of 145 cases using alpha-synuclein immunohistochemistry. Brain Pathol.

[8] Hashimoto M, Masliah E (1999). Alpha-synuclein in Lewy body disease and Alzheimer’s disease. Brain Pathol.

[9] Lippa CF, Fujiwara H, Mann DM, Giasson B, Baba M, Schmidt ML (1998). Lewy bodies contain altered alpha-synuclein in brains of many familial Alzheimer’s disease patients with mutations in presenilin and amyloid precursor protein genes. Am J Pathol.

[10] Arawaka S, Saito Y, Murayama S, Mori H (1998). Lewy body in neurodegeneration with brain iron accumulation type 1 is immunoreactive for alpha-synuclein. Neurology.

[11] Wakabayashi K, Yoshimoto M, Fukushima T, Koide R, Horikawa Y, Morita T (1999). Widespread occurrence of alpha-synuclein/NACP-immunoreactive neuronal inclusions in juvenile and adult-onset Hallervorden-Spatz disease with Lewy bodies. Neuropathol Appl Neurobiol.

[12] Newell KL, Boyer P, Gomez-Tortosa E, Hobbs W, Hedley-Whyte ET, Vonsattel JP (1999). Alpha-synuclein immunoreactivity is present in axonal swellings in neuroaxonal dystrophy and acute traumatic brain injury. J Neuropathol Exp Neurol.

[13] Wakabayashi K, Fukushima T, Koide R, Horikawa Y, Hasegawa M, Watanabe Y (2000). Juvenile-onset generalized neuroaxonal dystrophy (Hallervorden-Spatz disease) with diffuse neurofibrillary and lewy body pathology. Acta Neuropathol.

[14] Galvin JE, Giasson B, Hurtig HI, Lee VM, Trojanowski JQ (2000). Neurodegeneration with brain iron accumulation, type 1 is characterized by alpha-, beta-, and gamma-synuclein neuropathology. Am J Pathol.

[15] Neumann M, Adler S, Schlüter O, Kremmer E, Benecke R, Kretzschmar HA (2000). Alpha-synuclein accumulation in a case of neurodegeneration with brain iron accumulation type 1 (NBIA-1, formerly Hallervorden-Spatz syndrome) with widespread cortical and brainstem-type Lewy bodies. Acta Neuropathol.

[16] Anderson JP, Walker DE, Goldstein JM, de Laat R, Banducci K, Caccavello RJ (2006). Phosphorylation of Ser-129 is the dominant pathological modification of alpha-synuclein in familial and sporadic Lewy body disease. J Biol Chem.

[17] Fujiwara H, Hasegawa M, Dohmae N, Kawashima A, Masliah E, Goldberg MS (2002). alpha-Synuclein is phosphorylated in synucleinopathy lesions. Nat Cell Biol.

[18] Sacino AN, Brooks M, Thomas MA, McKinney AB, McGarvey NH, Rutherford NJ (2014). Amyloidogenic α-synuclein seeds do not invariably induce rapid, widespread pathology in mice. Acta Neuropathol.

[19] Waxman EA, Giasson BI (2008). Specificity and regulation of casein kinase-mediated phosphorylation of alpha-synuclein. J Neuropathol Exp Neurol.

[20] Luk KC, Kehm VM, Zhang B, O’Brien P, Trojanowski JQ, Lee VMY (2012). Intracerebral inoculation of pathological α-synuclein initiates a rapidly progressive neurodegenerative α-synucleinopathy in mice. J Exp Med.

[21] Luk KC, Kehm V, Carroll J, Zhang B, O’Brien P, Trojanowski JQ (2012). Pathological α-synuclein transmission initiates Parkinson-like neurodegeneration in nontransgenic mice. Science.

[22] Volpicelli-Daley LA, Luk KC, Patel TP, Tanik SA, Riddle DM, Stieber A (2011). Exogenous α-synuclein fibrils induce Lewy body pathology leading to synaptic dysfunction and neuron death. Neuron.

[23] Volpicelli-Daley LA, Gamble KL, Schultheiss CE, Riddle DM, West AB, Lee VM-Y (2014). Formation of α-synuclein Lewy neurite-like aggregates in axons impedes the transport of distinct endosomes. Mol Biol Cell.

[24] Abeliovich A, Schmitz Y, Fariñas I, Choi-Lundberg D, Ho WH, Castillo PE (2000). Mice lacking alpha-synuclein display functional deficits in the nigrostriatal dopamine system. Neuron.

[25] Giasson BI, Duda JE, Quinn SM, Zhang B, Trojanowski JQ, Lee VM-Y (2002). Neuronal alpha-synucleinopathy with severe movement disorder in mice expressing A53T human alpha-synuclein. Neuron.

[26] Rutherford NJ, Sacino AN, Brooks M, Ceballos-Diaz C, Ladd TB, Howard JK (2015). Studies of lipopolysaccharide effects on the induction of α-synuclein pathology by exogenous fibrils in transgenic mice. Mol Neurodegener.

[27] Sacino AN, Brooks M, McKinney AB, Thomas MA, Shaw G, Golde TE (2014). Brain injection of α-synuclein induces multiple proteinopathies, gliosis, and a neuronal injury marker. J Neurosci.

[28] Sacino AN, Brooks M, Thomas MA, McKinney AB, Lee S, Regenhardt RW (2014). Intramuscular injection of α-synuclein induces CNS α-synuclein pathology and a rapid-onset motor phenotype in transgenic mice. Proc Natl Acad Sci U S A.

[29] Zhu Q, Couillard-Després S, Julien JP (1997). Delayed maturation of regenerating myelinated axons in mice lacking neurofilaments. Exp Neurol.

[30] Giasson BI, Jakes R, Goedert M, Duda JE, Leight S, Trojanowski JQ (2000). A panel of epitope-specific antibodies detects protein domains distributed throughout human alpha-synuclein in Lewy bodies of Parkinson’s disease. J Neurosci Res.

[31] Giasson BI, Murray IV, Trojanowski JQ, Lee VM (2001). A hydrophobic stretch of 12 amino acid residues in the middle of alpha-synuclein is essential for filament assembly. J Biol Chem.

[32] Greenbaum EA, Graves CL, Mishizen-Eberz AJ, Lupoli MA, Lynch DR, Englander SW (2005). The E46K mutation in alpha-synuclein increases amyloid fibril formation. J Biol Chem.

[33] Emmer KL, Waxman EA, Covy JP, Giasson BI (2011). E46K human alpha-synuclein transgenic mice develop Lewy-like and tau pathology associated with age-dependent, detrimental motor impairment. J Biol Chem.

[34] Sacino AN, Thomas MA, Ceballos-Diaz C, Cruz PE, Rosario AM, Lewis J (2013). Conformational templating of α-synuclein aggregates in neuronal-glial cultures. Mol Neurodegener.

[35] Waxman EA, Giasson BI (2010). A novel, high-efficiency cellular model of fibrillar alpha-synuclein inclusions and the examination of mutations that inhibit amyloid formation. J Neurochem.

[36] Okochi M, Walter J, Koyama A, Nakajo S, Baba M, Iwatsubo T (2000). Constitutive phosphorylation of the Parkinson’s disease associated alpha-synuclein. J Biol Chem.

[37] Ishii A, Nonaka T, Taniguchi S, Saito T, Arai T, Mann D (2007). Casein kinase 2 is the major enzyme in brain that phosphorylates Ser129 of human alpha-synuclein: Implication for alpha-synucleinopathies. FEBS Lett.

[38] Link WT, Grant P, Hidaka H, Pant HC (1992). Casein kinases I and II from squid brain exhibit selective neurofilament phosphorylation. Mol Cell Neurosci.

[39] Link WT, Dosemeci A, Floyd CC, Pant HC (1993). Bovine neurofilament-enriched preparations contain kinase activity similar to casein kinase I--neurofilament phosphorylation by casein kinase I (CKI). Neurosci Lett.

[40] Nakamura Y, Hashimoto R, Kashiwagi Y, Wada Y, Sakoda S, Miyamae Y (1999). Casein kinase II is responsible for phosphorylation of NF-L at Ser-473. FEBS Lett.

[41] Xu ZS, Liu WS, Willard M (1990). Identification of serine 473 as a major phosphorylation site in the neurofilament polypeptide NF-L. J Neurosci.

[42] Waxman EA, Giasson BI (2011). Characterization of kinases involved in the phosphorylation of aggregated α-synuclein. J Neurosci Res.

[43] Mbefo MK, Paleologou KE, Boucharaba A, Oueslati A, Schell H, Fournier M (2010). Phosphorylation of synucleins by members of the Polo-like kinase family. J Biol Chem.

[44] Pronin AN, Morris AJ, Surguchov A, Benovic JL (2000). Synucleins are a novel class of substrates for G protein-coupled receptor kinases. J Biol Chem.

[45] Hara S, Arawaka S, Sato H, Machiya Y, Cui C, Sasaki A (2013). Serine 129 phosphorylation of membrane-associated α-synuclein modulates dopamine transporter function in a G protein-coupled receptor kinase-dependent manner. Mol Biol Cell.

[46] Zhang S, Xie J, Xia Y, Yu S, Gu Z, Feng R (2015). LK6/Mnk2a is a new kinase of alpha synuclein phosphorylation mediating neurodegeneration. Sci Rep.

[47] Inglis KJ, Chereau D, Brigham EF, Chiou S-S, Schöbel S, Frigon NL (2009). Polo-like kinase 2 (PLK2) phosphorylates alpha-synuclein at serine 129 in central nervous system. J Biol Chem.

[48] Julien JP, Mushynski WE (1998). Neurofilaments in health and disease. Prog Nucleic Acid Res Mol Biol.

[49] Josephs KA, Holton JL, Rossor MN, Braendgaard H, Ozawa T, Fox NC (2003). Neurofilament inclusion body disease: a new proteinopathy?. Brain.

[50] Mackenzie IRA, Feldman H (2004). Neurofilament inclusion body disease with early onset frontotemporal dementia and primary lateral sclerosis. Clin Neuropathol.

[51] Cairns NJ, Jaros E, Perry RH, Armstrong RA (2004). Temporal lobe pathology of human patients with neurofilament inclusion disease. Neurosci Lett.

[52] Josephs KA, Uchikado H, McComb RD, Bashir R, Wszolek Z, Swanson J (2005). Extending the clinicopathological spectrum of neurofilament inclusion disease. Acta Neuropathol.

[53] Uchikado H, Li A, Lin W-L, Dickson DW (2006). Heterogeneous inclusions in neurofilament inclusion disease. Neuropathol.

[54] Corbo M, Hays AP (1992). Peripherin and neurofilament protein coexist in spinal spheroids of motor neuron disease. J Neuropathol Exp Neurol.

[55] Manetto V, Sternberger NH, Perry G, Sternberger LA, Gambetti P (1988). Phosphorylation of neurofilaments is altered in amyotrophic lateral sclerosis. J Neuropathol Exp Neurol.

[56] Nakazato Y, Sasaki A, Hirato J, Ishida Y (1984). Immunohistochemical localization of neurofilament protein in neuronal degenerations. Acta Neuropathol.

[57] Itoh K, Negishi H, Obayashi C, Hayashi Y, Hanioka K, Imai Y (1993). Infantile neuroaxonal dystrophy--immunohistochemical and ultrastructural studies on the central and peripheral nervous systems in infantile neuroaxonal dystrophy. Kobe J Med Sci.

[58] Wu E, Dickson DW, Jacobson S, Raine CS (1993). Neuroaxonal dystrophy in HTLV-1-associated myelopathy/tropical spastic paraparesis: neuropathologic and neuroimmunologic correlations. Acta Neuropathol.

[59] Ksiezak-Reding H, Yen SH (1987). Two monoclonal antibodies recognize Alzheimer’s neurofibrillary tangles, neurofilament, and microtubule-associated proteins. J Neurochem.

[60] Schmidt ML, Lee VM, Trojanowski JQ (1990). Relative abundance of tau and neurofilament epitopes in hippocampal neurofibrillary tangles. Am J Pathol.

