# Supplementary figures and images for: Novel antibodies to phosphorylated α-synuclein serine 129 and NFL serine 473 demonstrate the close molecular homology of these epitopes
Source: Acta Neuropathol Commun. 2016 Aug 8;4:80. doi: 10.1186/s40478-016-0357-9 (PMC4977832; doi:10.1186/s40478-016-0357-9)

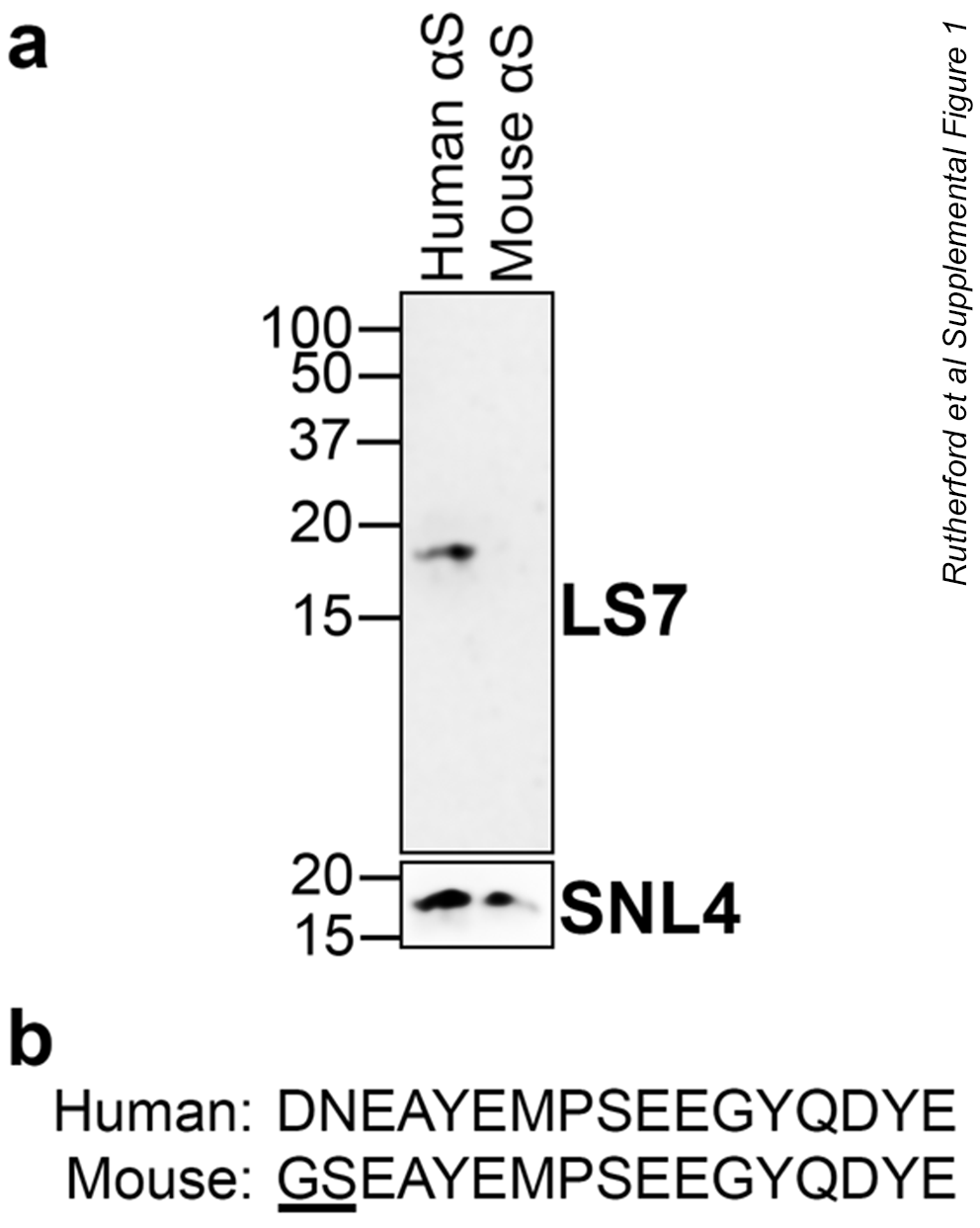

Supplement: Additional file 1: Figure S1. — Specificity of novel antibody LS7 for human αS. (a) Recombinant human and mouse αS (50 ng) were resolved onto 13 % polyacrylamide gels and analyzed by immunoblotting with LS7 and anti-αS antibody SNL-4 (residues 2–12 of αS). The mobility of molecular mass markers are shown on the left. (b) Sequence of the pSer129long peptide (human αS) with the corresponding mouse αS sequence underneath. The line indicates amino acids that are not shared. (TIF 216 kb) [file 40478_2016_357_MOESM1_ESM.tif]

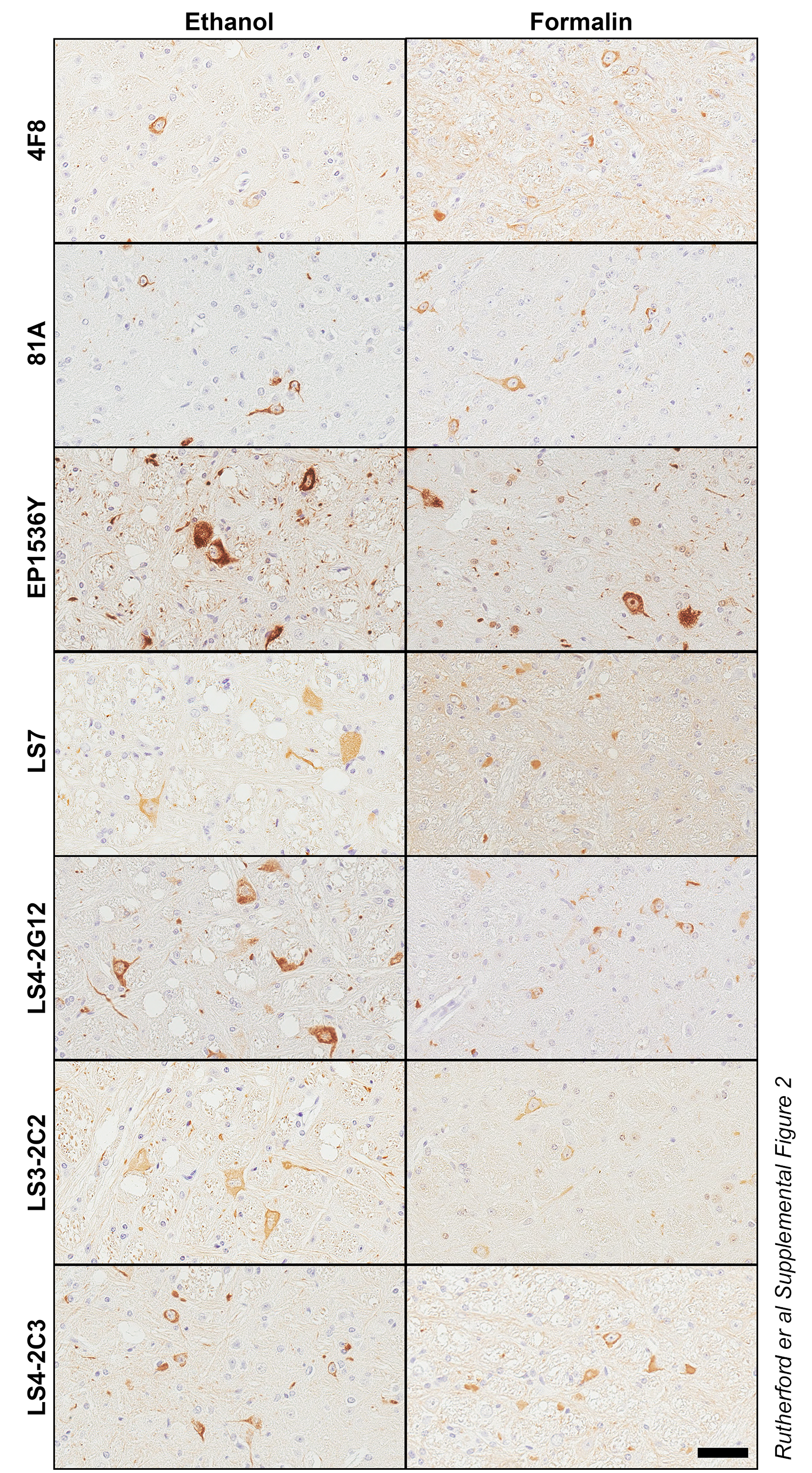

Supplement: Additional file 2: Figure S2. — IHC analyses showing antibody reactivity in formalin and ethanol fixed tissues. Representative IHC staining of the brainstem of M83 mice injected in the gastrocnemius muscle with αS fibrils. The tissue of one mouse (left) was fixed in 150 mM NaCl/70 % ethanol and the other (right) was fixed in formalin. All of the antibodies were able to stain pathology in both formalin and ethanol fixed tissue, however some staining appeared weaker in the formalin fixed tissue (LS4-2G12 and LS3-2C2). Scale bar = 50 μm. (TIF 9583 kb) [file 40478_2016_357_MOESM2_ESM.tif]

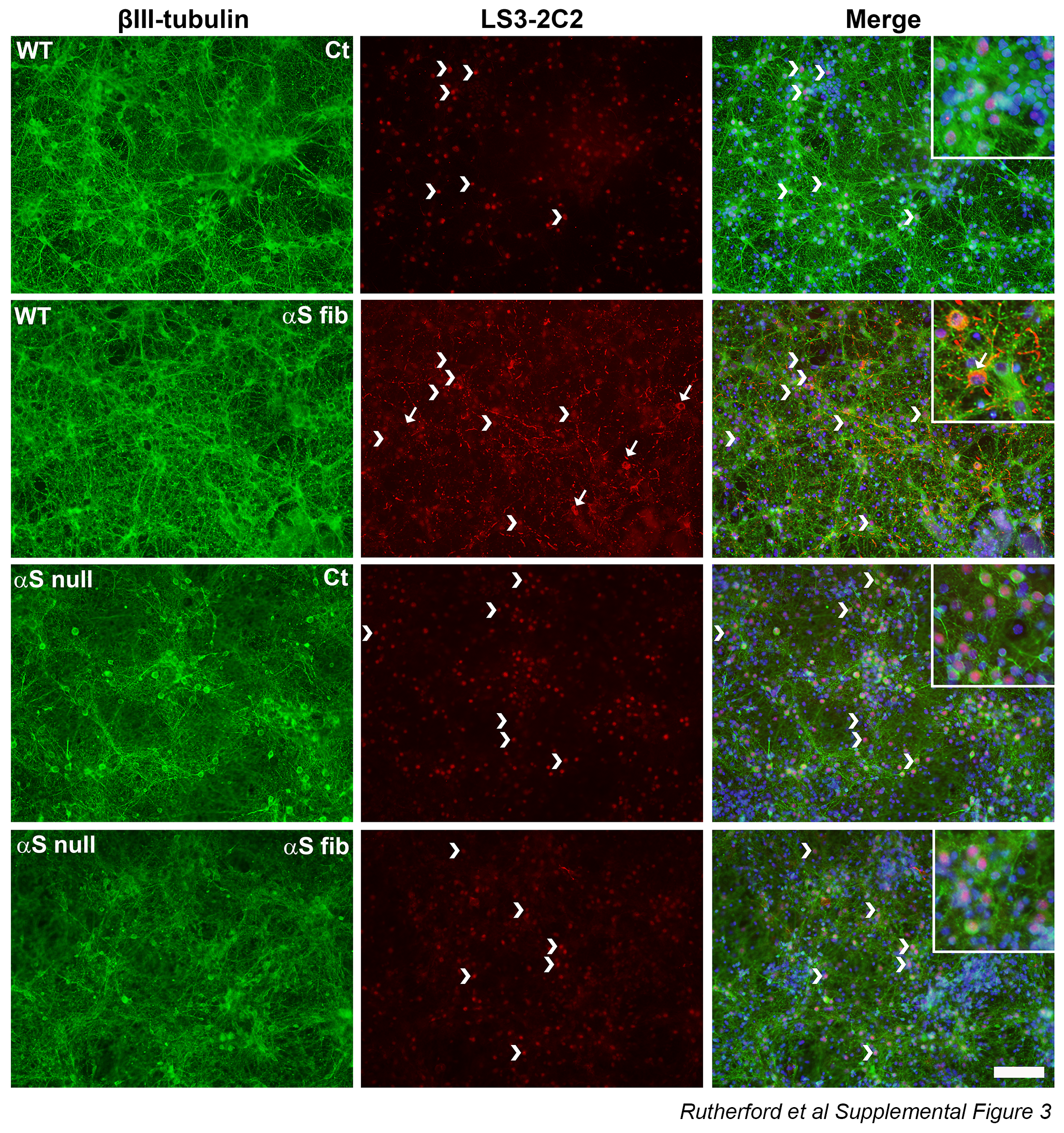

Supplement: Additional file 3: Figure S3. — Analysis of the induction of endogenous αS aggregates with exogenous αS mouse fibrils in primary neuronal-glial cultures using antibody LS3-2C2. Primary neuronal-glial cultures from WT mice or αS null mice were cultured for 6 days and either maintained without other treatment for 8 days (Ct) or treated with mouse αS fibrils (20 μg/ml; αS fib) for 8 days. Double immunofluorescence analysis with antibodies LS3-2C2 (red) and specific neuronal marker βIII-tubulin (green) was performed. Cells were also counterstained with DAPI and merged images are shown. Higher magnification merged images are shown on the far right. Arrows depict induced labeled αS aggregates and arrowheads depict nuclear staining. Bar = 100 μm and 250 μm for the higher magnification images on the right. (TIF 7508 kb) [file 40478_2016_357_MOESM3_ESM.tif]
